# Anesthetic Management of Atypical Toxic Epidermal Necrolysis in a Six-Month-Old Patient Undergoing Burn Surgery: A Case Report

**DOI:** 10.7759/cureus.83952

**Published:** 2025-05-12

**Authors:** Cassidy Chau, Anthony Q Dang, Thong Nguyen

**Affiliations:** 1 Anesthesiology and Perioperative Medicine, University of Texas Medical Branch at Galveston, Galveston, USA; 2 Biology, University of California Davis, Davis, USA

**Keywords:** burn resuscitation, fiberoptic intubation, ketamine and dexmedetomidine, pediatric burn pain management, toxic epidermal necrolysis (ten)

## Abstract

Toxic epidermal necrolysis (TEN) is a rare, life-threatening skin condition that involves widespread skin detachment and mucous membrane damage. We present a case of a six-month-old male with atypical TEN transferred from an outside hospital in Mexico to our facility. The patient had widespread full-thickness necrotic wounds but no bullae or sloughing. Since the diagnosis was unclear at the time, it posed a significant challenge to make an appropriate anesthetic plan given the need for prolonged intubation after extensive burn debridement and grafting surgery. This case report highlights the importance of perioperative assessment, induction strategy, airway management, resuscitation, and pain control in an atypical TEN patient.

## Introduction

Toxic epidermal necrolysis (TEN) is a rare, life-threatening skin condition involving widespread skin detachment and mucous membrane damage. The incidence of TEN is approximately 0.4 to 1.2 cases per million people per year. Although rare, it has a high mortality rate of about 25-30%, with almost all patients having to be admitted to the burn or intensive care unit [1]. Since patients with TEN have multi-organ involvement, including the airway, endotracheal intubation (ETT) and bag-masking are difficult. Fluid and blood resuscitation, in addition to electrolyte correction and pain control, are also critical components of supportive care in TEN. We present a case of a six-month-old male with atypical TEN who required ETT via fiberoptic nasotracheal route for surgery and prolonged pediatric intensive care unit (PICU) stay.

## Case presentation

A six-month-old, 7.7-kg male from Mexico presented with 38% total body surface area full-thickness necrotic wounds involving the upper and lower extremities, chest, face, buttocks, and penis. On December 1st, the patient was treated with paracetamol for fussiness and irritability. The next day, he developed a rash on his legs and abdomen, which was initially diagnosed as varicella. His mother was unable to recall the specific medications administered during treatment. After discharge, the patient experienced vomiting and lethargy, prompting hospitalization for suspected *Rickettsia* infection.

During his hospital stay, the patient developed widespread erythema and a progressive rash that transitioned from red to purple to black. He became hemodynamically unstable and experienced seizures, necessitating intubation and vasopressor support. After stabilization and subsequent extubation on December 4th, he was transferred to a pediatric hospital in Culiacan, Mexico, for management of a suspected meningococcal infection. The patient was isolated for 16 days before being transferred to our facility hospital.

**Figure 1:**
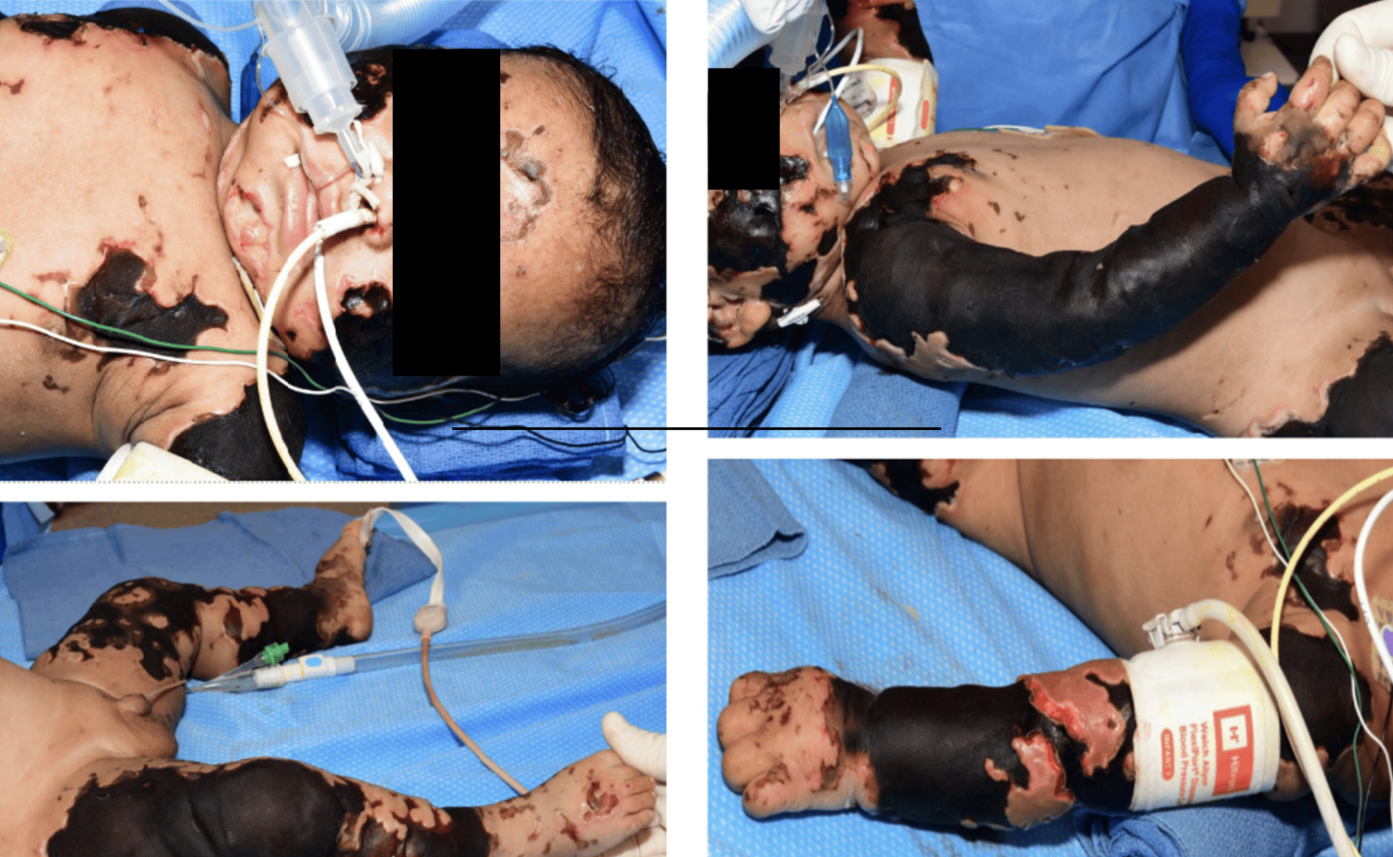
Images of the patient's full-thickness necrotic wounds The authors discussed and obtained consent from the parent(s) to use the patient's images/information for the purpose of education and research.

On arrival, the patient presented with full-thickness necrotic wounds, as shown in Figure 1. A comprehensive preoperative evaluation revealed a Mallampati score of II, a thyromental distance exceeding 5 cm, and a full neck range of motion. Given the complexity of the surgery and large volume fluid/blood resuscitation, prolonged intubation in the PICU was deemed essential.

Premedication included 1 mg of midazolam for anxiolysis and 0.1 mg of glycopyrrolate as an antisialagogue. Standard monitors were placed, and anesthesia was induced with 2 micrograms of dexmedetomidine (DEX) and intermittent ketamine boluses, titrated to a total dose of 5 mg/kg. Oral and nasal cavities were carefully suctioned to clear secretions, and oxymetazoline spray was applied bilaterally to reduce nasal mucosal swelling.

**Figure 2:**
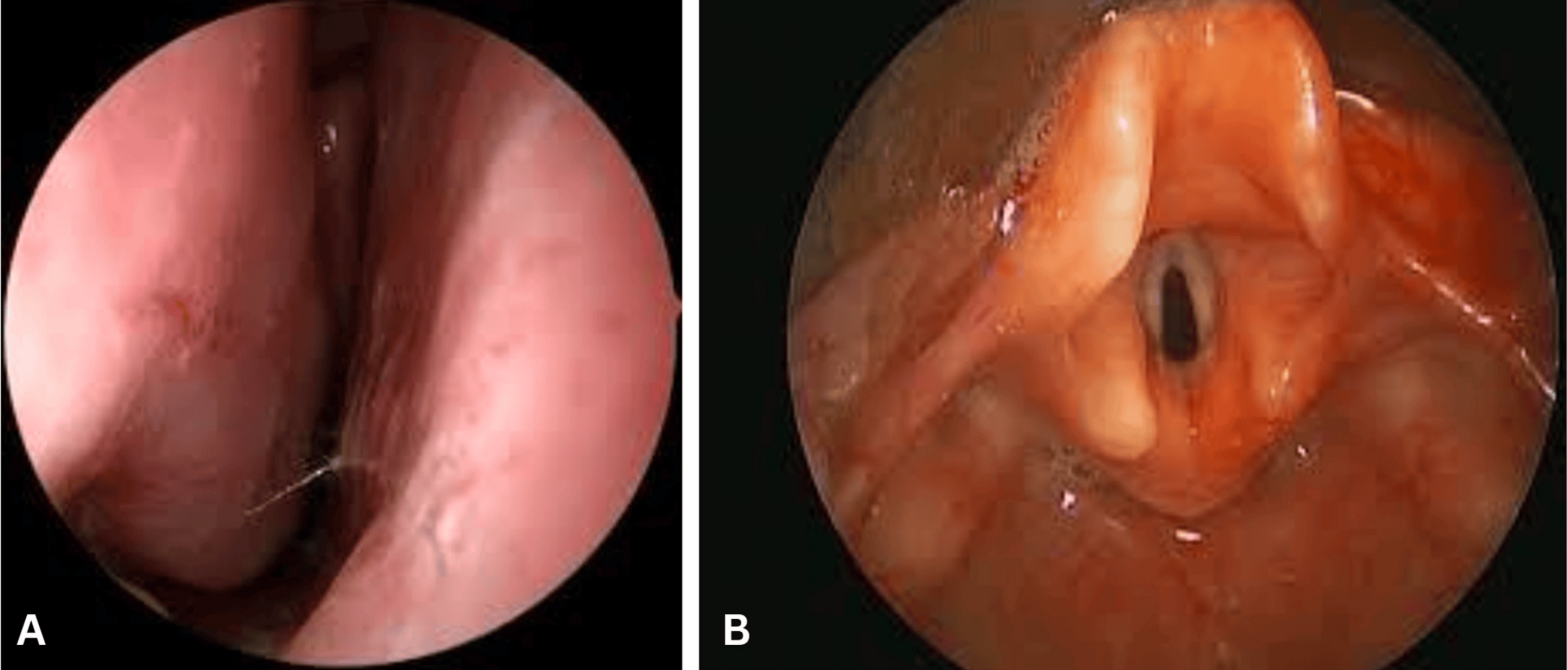
Fiberoptic examination of the patient's nasopharynx Fiberoptic examination demonstrating (A) normal right nasal septum with spotty erythema and (B) mild glottic swelling. Notably, no friability or mucosal bleeding was observed.

The patient remained spontaneously ventilating with a procedural oxygen mask. A 2.8-mm bronchoscope preloaded with a 4.0-mm ETT was advanced through the right nare, revealing a normal septum with spotty erythema (Figure 2A). A gentle Larson’s maneuver improved visualization of the laryngeal structures, revealing mild glottic swelling and inflammation without friable or bleeding mucosa (Figure 2B). Two milliliters of 2% lidocaine were then applied topically to the vocal cords for anesthesia. The bronchoscope was advanced into the trachea, and proper ETT placement was confirmed. The tube was carefully padded with soft gauze and secured with a septal tie.

During the surgery, anesthesia was maintained with 0.6 MAC of isoflurane, 1.5 mg of intravenous methadone, and intermittent boluses of fentanyl (max 10 mcg). The 5% dextrose, 0.9% normal saline was infused at 32 ml/hour as a maintenance fluid. The patient was resuscitated with 320 mL (~40 mL/kg) of leukocyte-reduced packed red blood cells and 150 mL (~20 mL/kg) of fresh frozen plasma. The estimated blood loss was 400 mL, and urine output was 80 mL. The surgery lasted for five hours, and no intraoperative complications occurred. Due to the large volume of resuscitation, the patient was kept intubated and sedated and returned to the pediatric ICU as planned.

## Discussion

Pathophysiology of TEN

TEN is a life-threatening skin disorder caused by an inappropriate immune response to certain medications. Common culprits include antibiotics (sulfonamides, chloramphenicol, penicillin, and quinolones), antiepileptics (barbiturates, carbamazepine, phenytoin, valproate, and lamotrigine), acetaminophen, NSAIDs, antiviral drugs (oseltamivir and abacavir), and allopurinol [2]. This disorder is immune-mediated, primarily involving T-cell activation in which the drug metabolites act as haptens, binding to keratinocyte proteins. This activates CD8+ cytotoxic T-cells and natural killer cells, inducing widespread keratinocyte apoptosis and epidermal detachment [3].

Differential diagnosis

In 90% of TEN cases, mucosal blistering precedes skin involvement [4]. However, this patient presented with widespread skin necrosis before any overt mucosal flaccid bullae. There was no skin/mucosal sloughing, phonation/breathing issue, or muffled voice. This presentation diverges from the classic pattern of TEN. TEN incidence in early infancy is extremely rare; only three well-documented cases have been reported [5]. Other differential diagnoses included cutaneous anthrax lesions, fulminant purpura secondary to meningococcal infection, or viral exanthem. From the patient's history, it is unclear whether any triggered medication(s). Interestingly, his skin biopsy confirmed the diagnosis of TEN.

Airway management options

Early tracheostomy is often used in cases of TEN where the mucosal membrane is damaged. A study of 40 patients with Stevens-Johnson syndrome/TEN was conducted between 2010 and 2015 to record intubation in such patients. Of the 43% who underwent early tracheostomy, 100% had oral involvement [6]. The typical approach for surgery would be to use oral ETT to prevent trauma or bleeding of the mucosa. In this case, nasotracheal intubation is preferred because of the lack of sloughing. Grensemann et al. reported that nasotracheal intubation was associated with fewer requirements of sedative medication, more assisted spontaneous breathing, and a higher degree of mobilization during physiotherapy [7].

Induction strategy

DEX and ketamine were induction agents of choice, aiming to maintain spontaneous ventilation while performing fiberoptic intubation in a potentially difficult airway. DEX, an alpha-2 agonist, has sedative, analgesic, and anxiolytic effects; has no respiratory depression; and has a short elimination half-life. Moreover, Lin et al. demonstrated that DEX is safe and effective for sedating pediatric burn patients on mechanical ventilation [8]. Adding DEX with ketamine promotes the advantage of balancing the hemodynamic and adverse effects of each other. DEX decreases the incidence of tachycardia, salivation, and hallucinations from ketamine, while ketamine prevents bradycardia and hypotension of DEX [9].

Analgesic strategy

Finally, methadone was selected for its potent analgesic effects and long duration of action, which is particularly beneficial in burn patients. Its NMDA receptor antagonist properties can help with neuropathic pain and opioid-induced hyperalgesia, both common in burn patients. It was also crucial to avoid triggering medications, including certain antibiotics, acetaminophen, and NSAIDs.

## Conclusions

This case highlights the complexity of managing a pediatric patient with atypical TEN. The fiberoptic evaluation and intubation show the importance of an individualized approach based on the patient's characteristics rather than relying solely on standard protocols. The proactive assessment of the oral and nasal mucosa, the cautious induction process to maintain spontaneous ventilation, and the strategic use of methadone for pain control collectively contributed to the positive surgical outcome. This case report underscores the need for ongoing research into the variability of TEN clinical presentations.
